# Factors associated with physician-reported treatment status of patients with osteoarthritis pain

**DOI:** 10.1186/s12891-022-05414-6

**Published:** 2022-05-26

**Authors:** Thomas J. Schnitzer, Rebecca L. Robinson, Leslie Tive, Joseph C. Cappelleri, Andrew G. Bushmakin, James Jackson, Mia Berry, Sophie Barlow, Chloe Walker, Lars Viktrup

**Affiliations:** 1 Northwestern University Feinberg School of Medicine, Chicago, IL USA; 2 Value, Evidence and Outcomes, Eli Lilly and Company, Lilly Research Labs, Lilly Corporate Center, Indianapolis, IN 46285 USA; 3 Internal Medicine, Global Medical Affairs, Pfizer Inc, New York, NY USA; 4 Statistical Research and Data Science Center, Pfizer Inc, New York, NY USA; 5 Real World Research, Adelphi Real World, Bollington, UK; 6 Neuroscience, Eli Lilly and Company, Indianapolis, IN USA

**Keywords:** Osteoarthritis, Pain, Prescription analgesic medication, Real-world clinical practice

## Abstract

**Background:**

Osteoarthritis (OA) is typically associated with pain, but many patients are not treated.

**Methods:**

This point in time study explored factors associated with treatment status, using logistic regression of data from the Adelphi OA Disease Specific Programme conducted in the United States. Patients’ treatment status was based on physician-reported, current: 1) prescription medication for OA vs. none; and 2) physician treatment (prescription medication and/or recommendation for specified nonpharmacologic treatment for OA [physical or occupational therapy, acupuncture, transcutaneous electrical nerve stimulation, or cognitive behavior therapy/psychotherapy]) vs. self-management (no prescription medication or specified nonpharmacologic treatment).

**Results:**

The 841 patients (including 57.0% knee OA, 31.9% hip OA) reported mild (45.4%) or moderate or severe (54.6%) average pain intensity over the last week. The majority were prescribed medication and/or recommended specified nonpharmacologic treatment; 218 were not prescription-medicated and 122 were self-managed. Bivariate analyses showed less severe patient-reported pain intensity and physician-rated OA severity, fewer joints affected by OA, lower proportion of joints affected by knee OA, better health status, lower body mass index, and lower ratings for cardiovascular and gastrointestinal risks, for those not prescribed medication (vs. prescription-medicated). Multivariate analyses confirmed factors significantly (*p* < 0.05) associated with prescription medication included (odds ratio): physician-rated current moderate OA severity (vs. mild, 2.03), patient-reported moderate OA severity 6 months ago (vs. mild, 1.71), knee OA (vs. not, 1.81), physician-recommended (0.28) and patient-reported (0.43) over-the-counter medication use (vs. not), prior surgery for OA (vs. not, 0.37); uncertain income was also significant. Factors significantly (*p* < 0.05) associated with physician treatment included (odds ratio): physician-recommended nonpharmacologic therapy requiring no/minimal medical supervision (vs. not, 2.21), physician-rated current moderate OA severity (vs. mild, 2.04), patient-reported over-the-counter medication use (vs. not, 0.26); uncertain time since diagnosis was also significant. Patient-reported pain intensity and most demographic factors were not significant in either model.

**Conclusions:**

Approximately 1 in 4 patients were not prescribed medication and 1 in 7 were self-managed, although many were using over-the-counter medications or nonpharmacologic therapies requiring no/minimal medical supervision. Multiple factors were significantly associated with treatment status, including OA severity and over-the-counter medication, but not pain intensity or most demographics.

**Supplementary Information:**

The online version contains supplementary material available at 10.1186/s12891-022-05414-6.

## Background

The pain and disability associated with osteoarthritis (OA) can limit activity and reduce quality of life [1]. Management may involve a comprehensive plan incorporating educational, behavioral, psychosocial, and physical interventions along with medications (acetaminophen, nonsteroidal anti-inflammatory drugs [NSAIDs], duloxetine, tramadol and other opioids, and intra-articular corticosteroids or hyaluronic acid) [2–4]. However, currently available medications may not have an acceptable benefit:risk profile [1], and are ineffective or unsuitable for some patients [2–4].

Patient-reported pain severity has been significantly associated with differences in treatment modalities, with patients with mild, moderate, and severe pain reported to be prescribed mostly nonpharmacologic therapy alone (23.8, 15.2, and 19.1%, respectively), prescription medication alone (15.7, 16.9, and 17.8%, respectively), or both (51.6, 63.9, and 59.9%, respectively); however, a sizable proportion of patients was prescribed neither (8.9, 4.0, and 3.2%, respectively) [5]. Many patients with knee OA do not receive core recommended nonsurgical treatments (pharmacotherapy for pain, exercise or physiotherapy, and weight loss if overweight or obese) before total knee arthroplasty [6]. Undertreatment of OA can result in the patient limiting their physical activity, which is counterproductive both for OA pain management and comorbidities such as obesity and cardiovascular disease [7].

Following discussions about the available options with their healthcare provider, patients with OA make treatment decisions taking into account treatment characteristics (including expectations, accessibility, and personalization), personal investment (financial and time), personal circumstances (age, body weight, comorbidities, previous experiences), and support network [8]. The aims of the current analyses were to describe the characteristics of patients with OA who are not prescription-medicated or who are self-managed, compared with those prescribed medication and/or recommended specified nonpharmacologic treatment, and to explore the factors associated with treatment status.

## Methods

This noninterventional, point-in-time (cross-sectional) survey of patients with OA and their physicians was based on data from the Adelphi Disease Specific Programme (DSP) for OA [9]. Data for the DSP were collected in the United States from February to May 2017. The DSP was performed in compliance with the United States Health Insurance Portability and Accountability Act 1996, and after review by the Western Institutional Review Board, the DSP methodology was granted ethical waiver. All patients provided informed consent.

### Study population

Primary care physicians, rheumatologists, and orthopedic surgeons were identified from public lists, and were eligible to participate in the DSP if they made treatment decisions for at least 10 patients with OA in a typical month. Participating physicians completed online patient record forms for their next 9 consecutive adult patients (≥18 years of age) diagnosed with OA. These patients were invited to participate in the DSP and those providing consent completed a questionnaire (paper-based) about their OA. To maintain real-world patterns in the DSP, diagnosis and OA severity were based on the physician’s judgment rather than specified criteria.

### Treatment status

Treatment definitions were based on physician report (from medical records or on the day of the study visit) to ensure a robust data set. Treatment status was defined based on physician-reported 1) current prescription of medication/s for OA (vs. none), and more broadly, 2) physician treatment (current prescription of medication/s for OA and/or physician recommendation/s for specified nonpharmacologic treatment for OA) vs. self-management (no prescription medication or specified nonpharmacologic treatment). Those patients with a current prescription for eligible medication/s for OA (documented in their medical record or prescribed on the day of the study visit) were designated Rx and all other patients were non-Rx. All Rx patients, plus those non-Rx patients with current recommendation/s for specified nonpharmacologic treatments (documented in their medical record or discussed on the day of the study visit) were designated physician-treated and the remainder were designated self-managed. All patients in the Rx group were included in the physician-treated group (Fig. S1).

Eligible prescribed medications included NSAIDs, opioids (including tramadol and nontramadol opioids), corticosteroids, viscosupplements, and others (including acetaminophen, capsaicin, glycosaminoglycans), with any route of administration. Specified nonpharmacologic treatments, recommended by the physician, were those that typically require prescription or referral from a physician and included physical/physiotherapy, occupational therapy, acupuncture, transcutaneous electrical nerve stimulation, or cognitive behavior therapy/psychotherapy.

Since there was no reliable mechanism to determine actual use, nonpharmacologic therapies that required no/minimal medical supervision (weight loss, fitness/exercise regimen, avoidance of painful activities, therapeutic massage, dietary supplements/home remedies, hypnosis, patient groups/forums, use of a walking stick/cane, use of a walker, use of a wheelchair, and ‘other’) were not considered in the determination of treatment status (if this was the patient’s only treatment, the patient was designated self-managed). Similarly, over-the-counter medications were not considered in the determination of treatment status (if this was the patient’s only medication they were in the non-Rx group, and in the absence of any specified nonpharmacologic treatments they were also self-managed).

### Measures

Physicians reported the patient’s age, sex, ethnicity, and insurance status, and patients reported their educational attainment and household income. Physicians also reported each patient’s body weight, height, presence of comorbidities (cardiologic, endocrine, neurologic/psychologic, respiratory, chronic low back pain, other musculoskeletal pain [including osteoporosis, neuropathic pain, rheumatoid arthritis, connective tissue disease, migraine, hemiplegia], cancer, obesity, and ‘other’ conditions), and rated their level of cardiovascular and gastrointestinal risks (both on scales of low, moderate, or high). Physicians also reported OA-specific measures including the date of the patient’s diagnosis, their rating of current OA severity (‘Please indicate below the severity of this patient’s osteoarthritis’ [currently], with possible responses mild, moderate, severe, or don’t know), the number and location of affected joints, and surgeries for OA (including previous surgeries and those planned for the future). Level of functional ability was rated by physicians in response to ‘Please rate your assessment of this patient’s functionality on a scale from 0 to 10? Where 0 = fully functional and 10 = completely impaired’. Physicians reported the patient’s OA treatments, including prescribed medications and recommendations for nonpharmacologic treatments and over-the-counter medications.

Patients scored their pain intensity using an 11-point numeric rating scale (0 = no pain, 10 = worst possible pain) in response to ‘How would you rate the intensity of your pain, on average, over the last week?’. Patients rated (mild, moderate, or severe) their OA severity 6 months previously, in response to ‘How severe would you rate your osteoarthritis condition?’. Patients completed the EQ-5D-5L to assess general health status on 5 dimensions (mobility, self-care, usual activities, pain/discomfort, and anxiety/depression) across 5 levels (no problems, slight problems, moderate problems, severe problems, and extreme problems), with higher index value indicating better health status [10]. Patients were asked about use (current, previous, never) of over-the-counter medications (‘Have you ever taken any medication that you have bought over the counter from a pharmacy or supermarket specifically to help treat your osteoarthritis symptoms?’).

### Statistical analyses

Analyses were conducted according to treatment status: 1) non-Rx vs. Rx, and 2) self-managed vs. physician-treated. All patients were included in both analyses.

Bivariate comparisons were conducted according to treatment status using Student’s t-test, chi-square, and Mann-Whitney [11]. In the contingency table analysis with an expected cell count of less than 5, Fisher’s exact test (for 2-by-2 tables) or Fisher’s generalized exact test was used (for r-by-c tables, where r or c or both exceed 2) [12].

Multivariate logistic regression was used to identify the factors significantly associated with treatment status. All those factors with two-tailed *p* < 0.25 [13, 14] in the bivariate analyses were included. The most important factors were identified using the LASSO (least absolute shrinkage and selection operator) approach with cross validation [15]. Covariates with nonzero coefficients (which have an impact on the outcome) identified by LASSO were included in the final logistic regression models, with *p* < 0.05 considered significant.

Data were managed and analyzed using SPSS version 7.5 (SPSS Inc., Chicago, IL, USA) and Stata version 17.0 (StataCorp, College Station, TX, USA).

## Results

The 153 physicians participating in the DSP included 81 primary care physicians, 35 rheumatologists, and 37 orthopedic surgeons (Table S1). The 841 patients with OA included in the analyses were mostly female (60.9%) and white/Caucasian (77.8%), with a mean age of 64.58 years (Table S2). Patient-rated average pain intensity over the last week was mild (45.4%), or moderate or severe (54.6%) (Table S2). The time since diagnosis of OA was < 6 months (47.0%), ≥6 months (4.8%), or uncertain (48.3%), and multiple joints were affected (mean 3.15), including 57.0% with knee OA and 31.9% with hip OA (Table S2). Almost all patients (99.5%) had insurance (Table S3).

### Prescription medication status (non-Rx vs. Rx)

Of the 841 patients, 25.9% (*n =* 218) were in the non-Rx group and 74.1% (*n =* 623) were in the Rx group (Fig. S1).

#### Bivariate analyses

Pain intensity differed significantly between groups (*p =* 0.0004), with moderate or severe average pain intensity over the last week reported by 42.7% of patients in the non-Rx group and 58.7% of patients in the Rx group (Table 1). Physician-rated current OA severity differed significantly between groups (*p* < 0.0001), with 41.9% of the non-Rx group and 68.6% of the Rx group having moderate or severe OA (Table 1). A similar pattern was seen for patient-rated OA severity 6 months ago (Table S3). Patients in the non-Rx group had fewer joints affected by OA (mean 2.66) compared with the Rx group (mean 3.32; *p =* 0.0006), and location of affected joints also differed, with joints other than a knee, hip, or back most frequently affected in the non-Rx group (50.9%) and a knee most frequently affected in the Rx group (60.2%) (Table 1). The non-Rx group reported better health status than the Rx group (*p* < 0.0001) (Table 1). Body mass index was lower in the non-Rx group (mean 27.64 kg/m^2^) compared with the Rx group (mean 29.07 kg/m^2^; *p =* 0.0018) (Table 1). Cardiovascular risk differed across the groups (*p =* 0.0068), with 34.9% (non-Rx) and 46.5% (Rx) of patients rated by physicians as moderate or high risk (Table S3). Gastrointestinal risk differed across the groups (*p =* 0.0185), with 26.1% (non-Rx) and 34.8% (Rx) of patients rated by physicians as moderate or high risk (Table S3). Cardiologic (55.0% vs. 69.0%; *p =* 0.0002) and endocrine (21.6% vs. 29.1%; *p* = 0.0322) comorbidities were less frequent in the non-Rx group compared with the Rx group, respectively (Table S3), although there was no difference in Charlson Comorbidity Index between groups (Table 1). There were few notable differences in demographics between the groups, with the exception of some variations in ethnicity (*p* = 0.0418) and patient responses to the income question (*p* = 0.0057) (Table 2).

**Table 1:** Clinical characteristics of patients with OA

	Prescription medication for OA^**a**^			Physician treatment for OA^**b**^		
	Non-Rx (***n =*** 218)	Rx (***n =*** 623)	***p***-value	Self-managed (***n =*** 122)	Physician-treated (***n =*** 719)	***p***-value
Time since OA diagnosis, *n* (%)			0.2161			0.1112
< 6 months	105 (48.2)	290 (46.5)		60 (49.2)	335 (46.6)	
6–12 months	3 (1.4)	24 (3.9)		0 (0.0)	27 (3.8)	
> 12 months	5 (2.3)	8 (1.3)		2 (1.6)	11 (1.5)	
Don’t know/missing	105 (48.2)	301 (48.3)		60 (49.2)	346 (48.1)	
Number of joints affected by OA, mean (SD)	2.66 (2.02)	3.32 (2.59)	0.0006	2.15 (1.60)	3.32 (2.55)	< 0.0001
Joints affected by OA, *n* (%)^c^						
Knee	104 (47.7)	375 (60.2)	0.0014	50 (41.0)	429 (59.7)	0.0001
Hip	59 (27.1)	209 (33.5)	0.077	23 (18.9)	245 (34.1)	0.0008
Back	66 (30.3)	246 (39.5)	0.0154	29 (23.8)	283 (39.4)	0.001
Other	111 (50.9)	292 (46.9)	0.3032	60 (49.2)	343 (47.7)	0.763
Body mass index (kg/m^2^), mean (SD)	27.64 (5.99)	29.07 (5.71)	0.0018	26.80 (4.87)	29.02 (5.90)	< 0.0001
Physician-rated current OA severity, *n* (%)^d^			< 0.0001			< 0.0001
Mild	126 (58.1)	195 (31.4)		78 (64.5)	243 (33.9)	
Moderate	71 (32.7)	330 (53.1)		36 (29.8)	365 (50.9)	
Severe	20 (9.2)	96 (15.5)		7 (5.8)	109 (15.2)	
Patient-reported average pain intensity over the last week, *n* (%)			0.0004			< 0.0001
Mild (0–3)	125 (57.3)	257 (41.3)		75 (61.5)	307 (42.7)	
Moderate (4–6)	58 (26.6)	244 (39.2)		34 (27.9)	268 (37.3)	
Severe (7–10)	35 (16.1)	122 (19.6)		13 (10.7)	144 (20.0)	
Health status: EQ-5D-5L index value, mean (SD)^e^	0.76 (0.26)	0.65 (0.29)	< 0.0001	0.80 (0.23)	0.66 (0.29)	< 0.0001
Functional assessment score, mean (SD)	3.21 (2.73)	4.21 (2.45)	< 0.0001	2.91 (2.83)	4.13 (2.48)	< 0.0001
Charlson Comorbidity Index, mean (SD)^f^	0.32 (0.66)	0.40 (0.81)	0.1817	0.25 (0.58)	0.40 (0.80)	0.0501

*p*-value for bivariate comparison (non-Rx vs. Rx; or self-managed vs. physician-treated)

^a^Current prescription medication

^b^Current prescription medication, and/or physician recommendation for physical or occupational therapy, acupuncture, transcutaneous electrical nerve stimulation, or cognitive behavior therapy/psychotherapy

^c^May be greater than 100% as multiple joints may be affected. ‘Other’ joints include hand/fingers, neck, shoulder, wrist, ankle, foot/toes, and elbow

^d^Sample size: *n =* 217 (non-Rx), *n =* 621 (Rx), *n =* 121 (self-managed), *n =* 717 (physician-treated)

^e^Sample size: *n =* 207 (non-Rx), *n =* 606 (Rx), *n =* 118 (self-managed), *n =* 695 (physician-treated)

^f^Assessment of comorbid burden [16]

*Non-Rx* not prescription-medicated, *OA* osteoarthritis, *Rx* prescription-medicated, *SD* standard deviation

**Table 2:** Demographics of patients with OA

	Prescription medication for OA^**a**^			Physician treatment for OA^**b**^		
	Non-Rx (***n =*** 218)	Rx (***n =*** 623)	***p***-value	Self-managed (***n =*** 122)	Physician-treated (***n =*** 719)	***p***-value
Age, years, mean (SD)	63.44 (12.21)	64.98 (11.52)	0.0949	62.22 (12.68)	64.98 (11.51)	0.0162
Range	21–90	30–90		21–90	25–90	
Sex, *n* (%)			0.5967			0.3912
Female	136 (62.4)	376 (60.4)		70 (57.4)	442 (61.5)	
Male	82 (37.6)	247 (39.6)		52 (42.6)	277 (38.5)	
Ethnicity, *n* (%)			0.0418			0.4729
White/Caucasian	176 (80.7)	478 (76.7)		103 (84.4)	551 (76.6)	
African American	19 (8.7)	75 (12.0)		8 (6.6)	86 (12.0)	
Native American	1 (0.5)	4 (0.6)		0 (0.0)	5 (0.7)	
Asian-Indian subcontinent	4 (1.8)	7 (1.1)		1 (0.8)	10 (1.4)	
Asian (other)	5 (2.3)	7 (1.1)		3 (2.5)	9 (1.3)	
Chinese	2 (0.9)	2 (0.3)		1 (0.8)	3 (0.4)	
Hispanic/Latino	7 (3.2)	45 (7.2)		6 (4.9)	46 (6.4)	
Middle Eastern	3 (1.4)	1 (0.2)		0 (0.0)	4 (0.6)	
Mixed race	1 (0.5)	4 (0.6)		0 (0.0)	5 (0.7)	
Annual household income, *n* (%)			0.0057			0.2443
≤ $50,000	25 (11.47)	119 (19.10)		14 (11.48)	130 (18.08)	
> $50,000 and ≤ $100,000	55 (25.23)	190 (30.50)		39 (31.97)	206 (28.65)	
> $100,000	50 (22.94)	115 (18.46)		21 (17.21)	144 (20.03)	
Prefer not to answer	65 (29.82)	129 (20.71)		35 (28.69)	159 (22.11)	
Missing	23 (10.55)	70 (11.24)		13 (10.66)	80 (11.13)	

*p*-value for bivariate comparison (non-Rx vs. Rx; or self-managed vs. physician-treated)

^a^Current prescription medication

^b^Current prescription medication, and/or physician recommendation for physical or occupational therapy, acupuncture, transcutaneous electrical nerve stimulation, or cognitive behavior therapy/psychotherapy

*Non-Rx* not prescription-medicated, *OA* osteoarthritis, *Rx* prescription-medicated, *SD* standard deviation

Both non-Rx and Rx groups included patients with recommendation/s for a wide range of nonpharmacologic treatments (Tables 3 and 4). Recommendations for physical/physiotherapy (37.2% vs. 44.9%; *p* = 0.0456) were less frequent, and recommendations for occupational therapy (10.1% vs. 5.3%; *p* = 0.0137) were more frequent, in the non-Rx group compared with the Rx group, respectively (Table 3). Recommendations for weight loss (36.2% vs. 46.2%; *p* = 0.0105) and use of a walking stick/cane (3.7% vs. 8.7%; *p* = 0.0151) were less frequent, and recommendations for dietary supplements/home remedies (9.2% vs. 4.7%; *p* = 0.0142) were more frequent, in the non-Rx group compared with the Rx group, respectively (Table 4). Over-the-counter medication use, based on both physician recommendation (64.7% vs. 31.5%; *p* < 0.0001) and patient report (60.2% vs. 35.1%; *p* < 0.0001), was more common in the non-Rx group compared with the Rx group, respectively (Table 4). The most frequently prescribed medication in the Rx group was an NSAID (Table 3).

**Table 3:** Physician-reported treatment modalities eligible for determination of treatment status

	Prescription medication for OA^**a**^			Physician treatment for OA^**b**^		
	Non-Rx (***n =*** 218)	Rx (***n =*** 623)	***p***-value	Self-managed (***n =*** 122)	Physician-treated (***n =*** 719)	***p***-value
Currently prescribed medication, *n* (%)						
Nonsteroidal anti-inflammatory drugs	0 (0.0)	484 (77.7)	–	0 (0.0)	484 (67.3)	–
Opioids	0 (0.0)	168 (27.0)	–	0 (0.0)	168 (23.4)	–
Corticosteroids	0 (0.0)	64 (10.3)	–	0 (0.0)	64 (8.9)	–
Viscosupplementation	0 (0.0)	23 (3.7)	–	0 (0.0)	23 (3.2)	–
Other^c^	0 (0.0)	173 (27.8)	–	0 (0.0)	173 (24.1)	–
Current nonpharmacologic treatment, *n* (%)						
Physical/physiotherapist	81 (37.2)	280 (44.9)	0.0456	0 (0.0)	361 (50.2)	–
Acupuncture	18 (8.3)	40 (6.4)	0.3571	0 (0.0)	58 (8.1)	–
Transcutaneous electrical nerve stimulation	12 (5.5)	38 (6.1)	0.7492	0 (0.0)	50 (7.0)	–
Cognitive behavior therapist/psychotherapist	0 (0.0)	10 (1.6)	0.0717	0 (0.0)	10 (1.4)	–
Occupational therapist	22 (10.1)	33 (5.3)	0.0137	0 (0.0)	55 (7.6)	–

*p*-value for bivariate comparison (non-Rx vs. Rx; or self-managed vs. physician-treated)

^a^Current prescription medication

^b^Current prescription medication, and/or physician recommendation for physical or occupational therapy, acupuncture, transcutaneous electrical nerve stimulation, or cognitive behavior therapy/psychotherapy

^c^Acetaminophen, capsaicin, glycosaminoglycans

*Non-Rx* not prescription-medicated, *OA* osteoarthritis, *Rx* prescription-medicated

**Table 4:** Treatment characteristics of patients with OA (not included in the determination of treatment status)

	Prescription medication for OA^**a**^			Physician treatment for OA^**b**^		
	Non-Rx (***n =*** 218)	Rx (***n =*** 623)	***p***-value	Self-managed (***n =*** 122)	Physician-treated (***n =*** 719)	***p***-value
Physician-reported current nonpharmacologic therapy, *n* (%)^c^						
Weight loss	79 (36.2)	288 (46.2)	0.0105	27 (22.1)	340 (47.3)	< 0.0001
Fitness/exercise regimen	109 (50.0)	325 (52.2)	0.5816	42 (34.4)	392 (54.5)	< 0.0001
Avoidance of painful activities	50 (22.9)	139 (22.3)	0.8492	15 (12.3)	174 (24.2)	0.0036
Therapeutic massage	26 (11.9)	81 (13.0)	0.6818	6 (4.9)	101 (14.0)	0.0051
Dietary supplements/home remedies	20 (9.2)	29 (4.7)	0.0142	3 (2.5)	46 (6.4)	0.0859
Hypnosis	0 (0.0)	1 (0.2)	1	0 (0.0)	1 (0.1)	1
Join patient groups/forums	0 (0.0)	4 (0.6)	0.5775	0 (0.0)	4 (0.6)	1
Walking stick/cane	8 (3.7)	54 (8.7)	0.0151	1 (0.8)	61 (8.5)	0.0027
Walker	4 (1.8)	17 (2.7)	0.4666	1 (0.8)	20 (2.8)	0.3428
Wheelchair	0 (0.0)	2 (0.3)	1	0 (0.0)	2 (0.3)	1
Other	12 (5.5)	7 (1.1)	0.0006	4 (3.3)	15 (2.1)	0.504
Physician-reported prior medication, *n* (%)			0.0012			0.0054
No	131 (60.1)	295 (47.4)		76 (62.3)	350 (48.7)	
Yes	87 (39.9)	328 (52.6)		46 (37.7)	369 (51.3)	
Physician-recommended current over-the-counter medication, *n* (%)			< 0.0001			0.0001
No	77 (35.3)	427 (68.5)		54 (44.3)	450 (62.6)	
Yes	141 (64.7)	196 (31.5)		68 (55.7)	269 (37.4)	
Patient-reported current over-the-counter medication, *n* (%)^d^			< 0.0001			< 0.0001
No	78 (39.8)	373 (64.9)		39 (36.4)	412 (62.0)	
Yes	118 (60.2)	202 (35.1)		68 (63.6)	252 (38.0)	
Prior surgery, *n* (%)			0.114			0.5237
No	162 (74.3)	495 (79.5)		98 (80.3)	559 (77.7)	
Yes	56 (25.7)	128 (20.5)		24 (19.7)	160 (22.3)	
Future surgery, *n* (%)			0.0074			0.0012
No	169 (77.5)	423 (67.9)		101 (82.8)	491 (68.3)	
Yes	49 (22.5)	200 (32.1)		21 (17.2)	228 (31.7)	

*p*-value for bivariate comparison (non-Rx vs. Rx; or self-managed vs. physician-treated)

^a^Current prescription medication

^b^Current prescription medication, and/or physician recommendation for physical or occupational therapy, acupuncture, transcutaneous electrical nerve stimulation, or cognitive behavior therapy/psychotherapy

^c^Nonpharmacologic therapies requiring no/minimal medical supervision (which were ineligible for the determination of treatment status). Physicians were asked ‘Which of the following suggestions have you made to the patient? [for the management of their osteoarthritis]’. See Table 3 for corresponding data for other response options that typically require prescription or referral from a physician (physical/physiotherapist, acupuncture, transcutaneous electrical nerve stimulation, cognitive behavior therapist/psychotherapist, occupational therapist)

^d^Sample size: *n =* 196 (non-Rx), *n =* 575 (Rx), *n =* 107 (self-managed), *n =* 664 (physician-treated)

*Non-Rx* not prescription-medicated, *OA* osteoarthritis, *Rx* prescription-medicated

#### Multivariate analyses

**Fig. 1:**
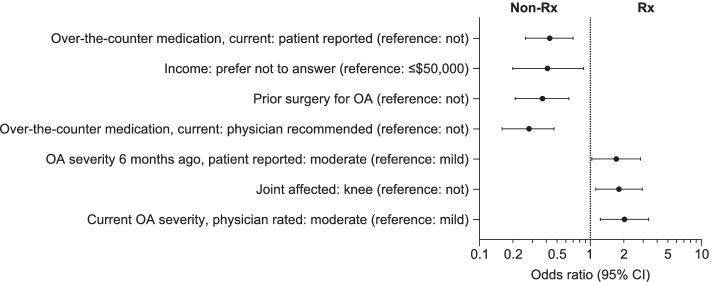
Factors associated with current prescription of medication/s for OA (non-Rx compared with Rx). Multivariate logistic regression. Logarithmic scale. Factors significantly associated with current prescription of medication/s for OA, non-Rx vs. Rx, *p* < 0.05. Constant: odds ratio 0.96 (95% CI: 0.19, 4.83). Log pseudolikelihood = − 320.77. Number of observations = 745. Wald chi^2^(24) = 160.22, Prob > chi^2^ = 0.00, Pseudo *R*^*2*^ = 0.24. For data, see Supplementary Table 4. *CI* confidence interval, *non-Rx* not prescription-medicated, *OA* osteoarthritis, *Rx* prescription-medicated

Patients in the non-Rx group were significantly more likely than patients in the Rx group to have OA of milder severity (physician-rated current and patient-reported 6 months ago), to have OA in joint/s other than a knee, to have undergone surgery for OA previously, to be recommended and using over-the-counter medication, and to prefer not to disclose their household income (Fig. 1). The odds of prescription medication were 2.03 times greater for those with OA severity rated as currently moderate by their physician (compared with mild OA), 1.71 times greater for patients reporting that their OA severity was moderate 6 months ago (compared with mild OA), and 1.81 times greater for patients with knee OA (compared with joints that were not a knee) (Table S4). The odds of prescription medication were 72% less for those with a current physician recommendation for over-the-counter medication (compared with no recommendation), 57% less for patients reporting current use of over-the-counter medications (compared with not), 63% less for those with prior surgery for OA (compared with none), and 59% less for those preferring not to answer the income question (compared with those reporting an income ≤$50,000) (Table S4).

### Physician treatment status (self-managed vs. physician-treated)

Of the 841 patients, 14.5% (*n =* 122) were self-managed and 85.5% (*n =* 719) were physician-treated (Fig. S1).

#### Bivariate analyses

Pain intensity differed significantly between groups (*p* < 0.0001), with moderate or severe average pain intensity over the last week reported by 38.5% of self-managed patients and 57.3% of physician-treated patients (Table 1). Physician-rated current OA severity differed significantly between groups (*p* < 0.0001), with 35.5% of the self-managed group and 66.1% of the physician-treated group having moderate or severe OA (Table 1). A similar pattern was seen for patient-rated OA severity 6 months ago (Table S3). Patients in the self-managed group had fewer joints affected by OA (mean 2.15) compared with the physician-treated group (mean 3.32; *p* < 0.0001), and location of affected joints also differed, with joints other than a knee, hip, or back most frequently affected in the self-managed group (49.2%) and a knee most frequently affected in the physician-treated group (59.7%) (Table 1). The self-managed group reported better health status than the physician-treated group (*p* < 0.0001) (Table 1). Body mass index was lower in the self-managed group (mean 26.80 kg/m^2^) compared with the physician-treated group (mean 29.02 kg/m^2^; *p* < 0.0001) (Table 1). There were few notable differences in demographics between the groups, although the self-managed group included younger patients compared with the physician-treated group (*p* = 0.0162) (Table 2).

The frequency of recommendations for nonpharmacologic therapies requiring no/minimal medical supervision (that were not included in the determination of treatment status) was lower in the self-managed group compared with the physician-treated group, including weight loss (22.1% vs. 47.3%; *p* < 0.0001), fitness/exercise regimen (34.4% vs. 54.5%; *p* < 0.0001), avoidance of painful activities (12.3% vs. 24.5%; *p* = 0.0036), therapeutic massage (4.9% vs. 14.0%; *p* = 0.0051), and use of a walking stick/cane (0.8% vs. 8.5%; *p* = 0.0027) (Table 4). Over-the-counter medication use, based on both physician recommendation (55.7% vs. 37.4%; *p* = 0.0001) and patient report (63.6% vs. 38.0%; *p* < 0.0001), was more common in the self-managed group compared with the physician-treated group, respectively (Table 4).

#### Multivariate analyses

**Fig. 2:**
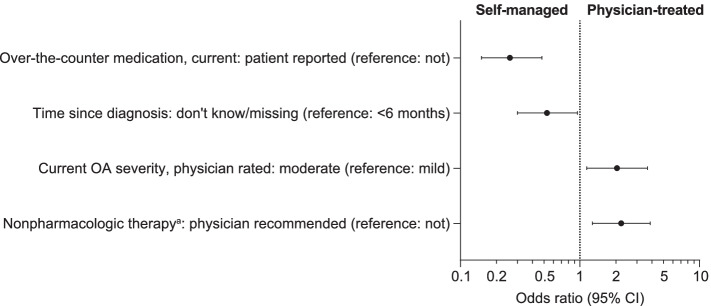
Factors associated with self-management vs. physician treatment for OA. Multivariate logistic regression. Logarithmic scale. Factors significantly associated with self-management vs. physician treatment, *p* < 0.05. Physician treatment defined as current prescription of medication and/or physician recommendation/s for specified nonpharmacologic treatment for OA (physical or occupational therapy, acupuncture, transcutaneous electrical nerve stimulation, or cognitive behavior therapy/psychotherapy). Self-management defined as no prescription medication or specified nonpharmacologic treatment. Constant: odds ratio 0.51 (95% CI: 0.11, 2.30). Log pseudolikelihood = − 226.31. Number of observations = 751. Wald chi^2^(26) = 156.00, Prob > chi^2^ = 0.00, Pseudo *R*^*2*^ = 0.25. ^a^Nonpharmacologic therapy requiring no/minimal medical supervision (which were ineligible for the determination of treatment status). For data, see Supplementary Table 5. *CI* confidence interval, *OA* osteoarthritis

Patients in the self-managed group were significantly more likely than patients in the physician-treated group to have no physician recommendation for nonpharmacologic therapy requiring no/minimal medical supervision, to have OA that the physician considered to be of milder severity, to be using over-the-counter medication, and to have an uncertain time since diagnosis (Fig. 2). The odds of physician treatment were 2.21 times greater for patients recommended nonpharmacologic therapy requiring no/minimal medical supervision (compared with no recommendation), and 2.04 times greater for patients with OA severity rated as currently moderate by their physician (compared with mild OA) (Table S5). The odds of physician treatment were 74% less for patients reporting current use of over-the-counter medications (compared with not), and 47% less for patients with an uncertain time since diagnosis (don’t know or missing, compared with diagnosis < 6 months ago) (Table S5).

## Discussion

This study found that, upon visiting a physician for OA, approximately 1 in 4 patients were not prescribed medication and 1 in 7 patients were self-managed, although many were using over-the-counter medications or nonpharmacologic therapies requiring no/minimal medical supervision. Multiple factors were significantly associated with treatment status, including physician-rated OA severity and use of over-the-counter medication.

Based on the bivariate analyses, patients who were not prescribed medication typically had less severe patient-reported pain intensity, less severe physician-rated OA severity, fewer joints affected by OA, lower proportion of joints affected by knee OA, better health status, lower body mass index, and lower ratings for cardiovascular and gastrointestinal risks, than prescription-medicated patients. After taking into consideration the multiple important factors identified by LASSO, the multivariate analyses confirmed that patients who were not prescription medicated were significantly more likely than prescription-medicated patients to have OA of milder severity, to have OA in joint/s other than a knee, to have undergone surgery for OA previously, and to be recommended and using over-the-counter medication. A previous study based on the same patient population found a significant association between treatment modalities and patient-reported pain [5]. Pain intensity was not significant in the current analyses after other factors were taken into account. However, symptoms are a factor when physicians determine OA severity [17]. Having medication prescribed was significantly associated with moderate OA severity (compared with mild) in the current study; conversely, severe OA (compared with mild) was not significant, although the sample size for patients with severe OA who were not prescription-medicated was relatively small. The significant association between having medication prescribed and knee OA may reflect the tendency for physicians to consider knees a lower priority for replacement than hips [18], among other factors. The significant association between not having medication prescribed and having had previous surgery for OA may indicate the success of surgery in alleviating pain, although patients could have learned better self-management skills after surgery, or it could indicate an unmet need for medication. There was no significant association with comorbidities, cardiovascular risk, or gastrointestinal risk in the multivariate analyses, suggesting that contraindications to medication were not important factors determining the presence or absence of prescription medication.

Based on the bivariate analyses, self-managed patients typically had less severe patient-reported pain intensity, less severe physician-rated OA severity, fewer joints affected by OA, lower proportion of joints affected by knee OA, better health status, lower body mass index, and younger age, than physician-treated patients. After taking into consideration the multiple important factors identified by LASSO, the multivariate analyses confirmed that patients in the self-managed group were significantly more likely than patients in the physician-treated group to have no physician recommendation for nonpharmacologic therapy requiring no/minimal medical supervision, to have OA that the physician considered to be of milder severity, to be using over-the-counter medication, and to have an uncertain time since diagnosis. The self-managed patients in the current study consulted a healthcare professional (about their OA, but not necessarily with respect to treatment), so they are a subset of the real-world non-treated population, who often do not seek help [19, 20].

Use of over-the-counter medication was significantly associated with treatment status. A previous study based on the same patient population found that the most frequently recommended over-the-counter medication was acetaminophen (51.8%), followed by ibuprofen (18.3%), naproxen (18.3%), glucosamine (6.4%), and other (5.2%); and that acetaminophen recommendation was more frequent in patients with more severe pain, and naproxen was more frequent in patients with less severe pain [5]; this may reflect a lack of efficacy of acetaminophen for OA [3, 4]. It is likely that when OA progresses to the extent that over-the-counter medications are no longer effective, patients may consult their healthcare provider for prescription medication.

Few demographic factors were significantly associated with treatment status in the current analyses. Previously, older age, male sex, and lower educational attainment were associated with less likelihood of having received recommended nonsurgical treatments prior to knee OA surgery [6]. However, this surgical cohort is not directly comparable with the patients in the current analyses.

Since many of the management strategies for OA are nonpharmacologic or available over-the-counter, this complicates observational studies of treatment, where it can be difficult to reliably determine what treatments are actually in use. Furthermore, some (such as exercise and weight loss) are discussed with almost all patients [5] and so are a background treatment recommendation by most definitions - but may be subject to variable compliance/actual use. The DSP methodology allowed a focus on physician records of what was prescribed and/or recommended, ensuring a robust data set. Self-managed OA was defined as no currently prescribed medication or physician recommendation for specified nonpharmacologic treatments that required medical supervision (physical or occupational therapy, acupuncture, transcutaneous electrical nerve stimulation, or cognitive behavior therapy/psychotherapy). Due to their requirement for medical supervision, the recommendations for these nonpharmacologic therapies were reliably documented in the medical records, and the likelihood of their actual use was high (although not measured by the current study). In contrast, other nonpharmacologic therapies that required no/minimal medical supervision, and over the counter medications, were not eligible for determining treatment status for the current analyses, since both the documentation of recommendation and data regarding the actual use of such therapies were likely less reliable. These decisions followed a previous analysis, based on the same patient population, that used a wider definition of treatment (including therapies requiring no/minimal supervision) that reported that 6.1% of patients received no treatment [5]. Increased medical supervision (via digital technology or physician assistance) and determination of the amount of supervision (via specific survey items) would facilitate the robust incorporation of these nonpharmacologic therapies requiring no/minimal medical supervision into definitions of treatment status in future studies.

This study has some limitations. The analyses were conducted for current treatment status and did not include previous treatments. Data were collected in the United States from participating primary care physicians, rheumatologists, and orthopedic surgeons; generalization to other countries and other specialties may not be possible. The patient population reflects those patients with OA consulting a healthcare professional and who were willing to take part in the study, which may not be generalizable. It is not possible to infer causality between factors and treatment status from the data set. Some confounding factors may not be accounted for, for example, influences of patients giving up on treatment, and although almost all patients in the current study were insured, out of pocket costs or other factors such as accessibility could have influenced treatment status. Physicians were instructed to collect data on consecutive patients to minimize selection bias.

## Conclusions

This study found that approximately 1 in 4 patients with OA were not prescribed medication and 1 in 7 patients self-managed their OA. The use of over-the-counter medication or nonpharmacologic therapies requiring no/minimal medical supervision was common. Patients who were not prescription-medicated were significantly more likely than prescription-medicated patients to have OA of milder severity, to have OA in joint/s other than a knee, to have undergone surgery for OA previously, to be recommended and using over-the-counter medication, and to prefer not to disclose their household income. Patients who were self-managed were significantly more likely than physician-treated patients to have no physician recommendation for nonpharmacologic therapy requiring no/minimal medical supervision, to have OA that the physician considered to be of milder severity, to be using over-the-counter medication, and to have an uncertain time since diagnosis.

## Supplementary Information

**Additional file 1.**

## Data Availability

The data that support the findings of this study are available from Adelphi Real World, but restrictions apply to the availability of these data, which were used under license for the current study and so are not publicly available. However, data are available from the authors upon reasonable request and with permission from Adelphi Real World.

## Abbreviations

DSP: Disease Specific Programme
LASSO: Least absolute shrinkage and selection operator
non-Rx: Not prescription-medicated
NSAID: Nonsteroidal anti-inflammatory drug
OA: Osteoarthritis
Rx: Prescription-medicated
