# Supplementary material for: Factors associated with physician-reported treatment status of patients with osteoarthritis pain
Source: BMC Musculoskelet Disord. 2022 May 26;23:498. doi: 10.1186/s12891-022-05414-6 (PMC9134575; doi:10.1186/s12891-022-05414-6)
Supplement: Supplementary file 1 — Additional file 1. [file 12891_2022_5414_MOESM1_ESM.docx]

Table S1. Physician characteristics

|  | **Total**  **(*N* = 153)** | **Orthopedic surgeon**  **(*n* = 37)** | **Rheumatologist**  **(*n* = 35)** | **Primary care physician**  **(*n* = 81)** |
| --- | --- | --- | --- | --- |
| Male | 111 (72.5) | 37 (100.0) | 26 (74.3) | 48 (59.3) |
| Years since specialty qualification |  |  |  |  |
| <4 | 9 (5.9) | 7 (18.9) | 0 (0.0) | 2 (2.5) |
| 4–14 | 25 (16.3) | 6 (16.2) | 8 (22.9) | 11 (13.6) |
| 15–24 | 46 (30.1) | 13 (35.1) | 8 (22.9) | 25 (30.9) |
| ≥25 | 73 (47.7) | 11 (29.7) | 19 (54.3) | 43 (53.1) |
| Region |  |  |  |  |
| Northeast | 47 (30.7) | 17 (45.9) | 10 (28.6) | 20 (24.7) |
| Midwest | 38 (24.8) | 8 (21.6) | 9 (25.7) | 21 (25.9) |
| South | 34 (22.2) | 6 (16.2) | 9 (25.7) | 19 (23.5) |
| West | 34 (22.2) | 6 (16.2) | 7 (20.0) | 21 (25.9) |
| Proportion of patients seen in setting, mean % (SD) |  |  |  |  |
| Private hospital | 5.5 (12.7) | 9.4 (14.9) | 3.4 (5.5) | 4.7 (13.5) |
| Public hospital | 6.1 (15.0) | 17.2 (25.3) | 2.1 (4.7) | 2.8 (7.5) |
| Private office | 79.2 (31.5) | 67.8 (33.1) | 91.5 (14.6) | 79.1 (34.3) |
| Public office | 8.0 (21.9) | 4.9 (9.4) | 2.7 (8.0) | 11.8 (28.5) |
| Other | 1.1 (8.4) | 0.7 (4.1) | 0.3 (1.2) | 1.7 (11.2) |
| Proportion of patients by physician-reported OA severity, mean % (SD) |  |  |  |  |
| Mild | 34.6 (16.5) | 33.9 (13.9) | 24.7 (12.5) | 39.3 (17.4) |
| Moderate | 41.6 (12.7) | 40.6 (11.7) | 47.3 (11.5) | 39.6 (13.1) |
| Severe | 23.7 (11.5) | 25.5 (10.8) | 28.0 (10.9) | 21.1 (11.4) |
| Routinely follow OA treatment guidelines | 70 (45.8) | 20 (54.1) | 11 (31.4) | 39 (48.1) |

Data are *n* (%) unless otherwise indicated

Adapted from table previously published under Creative Commons Attribution-Non Commercial 4.0 International License (<http://creativecommons.org/licenses/by-nc/4.0/>) in: Robinson RL, Schnitzer TJ, Barlow S, Berry M, Bushmakin AG, Cappelleri JC, Tive L, Jackson J, Jackson J, Viktrup L. Satisfaction with medications prescribed for osteoarthritis: a cross-sectional survey of patients and their physicians in the United States. Pain Ther. 2022;11(1):191-208. doi: 10.1007/s40122-021-00350-0

*OA* osteoarthritis, *SD* standard deviation

Table S2. Characteristics of all patients with OA included in the study

|  | **All patients** |
| --- | --- |
|  | ***N* = 841** |
| Age, years, mean (SD) | 64.58 (11.72) |
| Female, *n* (%) | 512 (60.9) |
| Ethnicity, *n* (%) |  |
| White/Caucasian | 654 (77.8) |
| African American | 94 (11.2) |
| Native American | 5 (0.6) |
| Asian-Indian subcontinent | 11 (1.3) |
| Asian (other) | 12 (1.4) |
| Chinese | 4 (0.5) |
| Hispanic/Latino | 52 (6.2) |
| Middle Eastern | 4 (0.5) |
| Mixed race | 5 (0.6) |
| Insurance, *n*/*n*^a^ |  |
| Insured/not insured | 829/4 |
| Time since OA diagnosis |  |
| Years, mean (SD)^b^ | 2.32 (3.79) |
| Categories, *n* (%) |  |
| <6 months | 395 (47.0) |
| ≥6 months | 40 (4.8) |
| Don't know/missing | 406 (48.3) |
| Number of joints affected by OA, mean (SD) | 3.15 (2.47) |
| Joints affected by OA, *n* (%)^c^ |  |
| Knee | 479 (57.0) |
| Hip | 268 (31.9) |
| Back | 312 (37.1) |
| Other | 403 (47.9) |
| Patient-reported average pain intensity over the last week, *n* (%) |  |
| Mild (0–3) | 382 (45.4) |
| Moderate (4–6) | 302 (35.9) |
| Severe (7–10) | 157 (18.7) |

^a^Sample size: *n =* 833

^b^Sample size: *n =* 435

^c^May be greater than 100% as multiple joints may be affected. ‘Other’ joints include hand/fingers, neck, shoulder, wrist, ankle, foot/toes, and elbow

*OA* osteoarthritis, *SD* standard deviation

Table S3. Characteristics of patients with OA

|  | **Prescription medication for OA^a^** | | | **Physician treatment for OA^b^** | | |
| --- | --- | --- | --- | --- | --- | --- |
|  | **Non-Rx  (*n =* 218)** | **Rx  (*n =* 623)** | ***p*-value** | **Self-managed  (*n =* 122)** | **Physician-treated  (*n =* 719)** | ***p*-value** |
| Insurance status, *n*/*n*^c^ |  |  |  |  |  |  |
| Insured/not insured | 215/1 | 614/3 | - | 121/0 | 708/4 | - |
| Insurance status, *n* (%)^c^ |  |  |  |  |  |  |
| Not Medicare | 130 (60.2) | 320 (51.9) | 0.0347 | 76 (62.8) | 374 (52.5) | 0.0359 |
| Medicare | 86 (39.8) | 297 (48.1) |  | 45 (37.2) | 338 (47.5) |  |
| Education, *n*/*n*/*n*^d^ |  |  |  |  |  |  |
| <High school/high school/>high school | 4/44/169 | 22/154/435 | 0.1235 | 3/27/92 | 23/171/512 | 0.8301 |
| Comorbidities, *n* (%) |  |  |  |  |  |  |
| Cardiologic | 120 (55.0) | 430 (69.0) | 0.0002 | 63 (51.6) | 487 (67.7) | 0.0005 |
| Endocrine | 47 (21.6) | 181 (29.1) | 0.0322 | 22 (18.0) | 206 (28.7) | 0.0147 |
| Neurologic/psychologic | 57 (26.1) | 199 (31.9) | 0.1095 | 27 (22.1) | 229 (31.8) | 0.031 |
| Respiratory conditions | 21 (9.6) | 51 (8.2) | 0.5111 | 13 (10.7) | 59 (8.2) | 0.3712 |
| Chronic low back pain | 24 (11.0) | 90 (14.4) | 0.202 | 10 (8.2) | 104 (14.5) | 0.0615 |
| Musculoskeletal pain | 32 (14.7) | 107 (17.2) | 0.3931 | 11 (9.0) | 128 (17.8) | 0.0157 |
| Cancer | 6 (2.8) | 20 (3.2) | 0.7367 | 2 (1.6) | 24 (3.3) | 0.4088 |
| Obesity | 21 (9.6) | 58 (9.3) | 0.888 | 5 (4.1) | 74 (10.3) | 0.0301 |
| Other | 32 (14.7) | 110 (17.7) | 0.3125 | 17 (13.9) | 125 (17.4) | 0.3468 |
| Cardiovascular risk, *n* (%) |  |  |  |  |  |  |
| Low | 142 (65.1) | 333 (53.5) | 0.0068 | 86 (70.5) | 389 (54.1) | 0.0007 |
| Moderate | 62 (28.4) | 253 (40.6) |  | 32 (26.2) | 283 (39.4) |  |
| High | 14 (6.4) | 37 (5.9) |  | 4 (3.3) | 47 (6.5) |  |
| Gastrointestinal risk, *n* (%) |  |  |  |  |  |  |
| Low | 161 (73.9) | 406 (65.2) | 0.0185 | 97 (79.5) | 470 (65.4) | 0.0021 |
| Moderate/high | 57 (26.1) | 217 (34.8) |  | 25 (20.5) | 249 (34.6) |  |
| Patient-reported OA severity 6 months ago, *n* (%)^e^ |  |  |  |  |  |  |
| Mild | 107 (51.7) | 165 (27.3) | <0.0001 | 67 (58.3) | 205 (29.5) | <0.0001 |
| Moderate | 79 (38.2) | 348 (57.6) |  | 40 (34.8) | 387 (55.6) |  |
| Severe | 21 (10.1) | 91 (15.1) |  | 8 (7.0) | 104 (14.9) |  |

*p*-value for bivariate comparison (non-Rx vs. Rx; or self-managed vs. physician-treated)

^a^Current prescription medication

^b^Current prescription medication, and/or physician recommendation for physical or occupational therapy, acupuncture, transcutaneous electrical nerve stimulation, or cognitive behavior therapy/psychotherapy

^c^Sample size: *n =* 216 (non-Rx), *n =* 617 (Rx), *n =* 121 (self-managed), *n =* 712 (physician-treated)

^d^ Sample size: *n =* 217 (non-Rx), *n =* 611 (Rx), *n =* 122 (self-managed), *n =* 706 (physician-treated)

^e^ Sample size: *n =* 207 (non-Rx), *n =* 604 (Rx), *n =* 115 (self-managed), *n =* 696 (physician-treated)

*Non-Rx* not prescription-medicated, *OA* osteoarthritis, *Rx* prescription-medicated

Table S4. Factors associated with current prescription of medication/s for OA (non-Rx compared with Rx)

|  | **Odds ratio** | **95% CI** | | ***p*-value** |
| --- | --- | --- | --- | --- |
| **Factors significantly associated with outcome in the final model** | | | | |
| Current OA severity, physician-rated: moderate (reference: mild) | 2.03 | 1.23 | 3.34 | 0.005 |
| Joint affected: knee (reference: not) | 1.81 | 1.12 | 2.94 | 0.016 |
| OA severity 6 months ago, patient-reported: moderate (reference: mild) | 1.71 | 1.03 | 2.83 | 0.036 |
| Over-the-counter medication, current: physician recommended (reference: not) | 0.28 | 0.16 | 0.47 | 0.000 |
| Prior surgery for OA (reference: not) | 0.37 | 0.21 | 0.64 | 0.000 |
| Income: prefer not to answer (reference: ≤$50,000) | 0.41 | 0.20 | 0.87 | 0.019 |
| Over-the-counter medication, current: patient-reported (reference: not) | 0.43 | 0.26 | 0.70 | 0.001 |
| **Factors not significantly associated with outcome in the final model** | | | | |
| Insurance status: Medicare (reference: not) | 1.19 | 0.77 | 1.83 | 0.427 |
| Income: >$50,000 and ≤$100,000 (reference: ≤$50,000) | 0.74 | 0.35 | 1.60 | 0.451 |
| Income: >$100,000 (reference: ≤$50,000) | 0.85 | 0.36 | 2.00 | 0.714 |
| Income: missing (reference: ≤$50,000) | 1.11 | 0.34 | 3.64 | 0.862 |
| Body mass index | 1.03 | 0.98 | 1.07 | 0.284 |
| Number of joints affected | 1.09 | 0.96 | 1.23 | 0.201 |
| Joint affected: back (reference: not) | 1.50 | 0.94 | 2.40 | 0.092 |
| Comorbidity: cardiologic (reference: not) | 1.45 | 0.91 | 2.33 | 0.121 |
| Cardiovascular risk: moderate (reference: low) | 1.16 | 0.69 | 1.95 | 0.564 |
| Cardiovascular risk: high (reference: low) | 0.62 | 0.23 | 1.66 | 0.338 |
| Prior medication, yes, physician-reported (reference: no) | 0.96 | 0.53 | 1.76 | 0.902 |
| Opioid use, ever, physician-reported (reference: not) | 2.29 | 0.80 | 6.56 | 0.123 |
| Functional assessment score, physician-rated | 1.09 | 0.98 | 1.21 | 0.121 |
| Average pain intensity over the last week, patient-reported: moderate 4–6 (reference: mild 0–3) | 1.08 | 0.61 | 1.91 | 0.804 |
| Average pain intensity over the last week, patient-reported: severe 7–10 (reference: mild 0–3) | 0.67 | 0.30 | 1.50 | 0.329 |
| Current OA severity, physician-rated: severe (reference: mild) | 2.23 | 0.96 | 5.22 | 0.063 |
| OA severity 6 months ago, patient-reported: severe (reference: mild) | 1.48 | 0.66 | 3.31 | 0.336 |

Multivariate logistic regression, non-Rx vs. Rx. The table includes all factors identified as important by LASSO. *p-*value indicates level of significance in the final model. Constant: odds ratio 0.96 (95% CI: 0.19, 4.83). Log pseudolikelihood = –320.77. Number of observations = 745. Wald chi^2^(24) = 160.22, Prob > chi^2^ = 0.00, Pseudo R^2^ = 0.24

*Non-Rx* not prescription-medicated, *CI* confidence interval, *LASSO* least absolute shrinkage and selection operator, *OA* osteoarthritis, *Rx* prescription-medicated

Table S5. Factors associated with self-management vs. physician treatment for OA

|  | **Odds Ratio** | **95% CI** | | ***p*-value** |
| --- | --- | --- | --- | --- |
| **Factors significantly associated with outcome in the final model** | | | | |
| Nonpharmacologic therapy^a^: physician recommended (reference: not) | 2.21 | 1.27 | 3.87 | 0.005 |
| Current OA severity, physician-rated: moderate (reference: mild) | 2.04 | 1.14 | 3.67 | 0.017 |
| Time since diagnosis: don't know/missing (reference: <6 months) | 0.53 | 0.30 | 0.95 | 0.033 |
| Over-the-counter medication, current: patient-reported (reference: not) | 0.26 | 0.15 | 0.48 | 0.000 |
| **Factors not significantly associated with outcome in the final model** | | | | |
| Ethnicity: African American (reference: white/Caucasian) | 1.75 | 0.57 | 5.40 | 0.331 |
| Ethnicity: Asian and ethnic minority (reference: white/Caucasian) | 1.20 | 0.50 | 2.87 | 0.680 |
| Income: >$50,000 and ≤$100,000 (reference: ≤$50,000) | 0.71 | 0.30 | 1.68 | 0.435 |
| Income: >$100,000 (reference: ≤$50,000) | 1.72 | 0.61 | 4.85 | 0.303 |
| Income: prefer not to answer (reference: ≤$50,000) | 0.67 | 0.28 | 1.61 | 0.367 |
| Income: missing (reference: ≤$50,000) | 0.95 | 0.26 | 3.47 | 0.937 |
| Body mass index | 1.04 | 0.99 | 1.09 | 0.148 |
| Time since diagnosis: ≥6 months (reference: <6 months) | 2.27 | 0.25 | 20.78 | 0.469 |
| Number of joints affected | 1.20 | 0.98 | 1.48 | 0.082 |
| Joint affected: knee (reference: not) | 1.69 | 0.99 | 2.87 | 0.053 |
| Joint affected: hip (reference: not) | 1.41 | 0.70 | 2.81 | 0.333 |
| Joint affected: back (reference: not) | 1.44 | 0.83 | 2.50 | 0.192 |
| Charlson Comorbidity Index | 1.11 | 0.68 | 1.81 | 0.676 |
| Comorbidity: cardiologic (reference: not) | 1.09 | 0.58 | 2.06 | 0.784 |
| Comorbidity: endocrine (reference: not) | 1.31 | 0.70 | 2.46 | 0.405 |
| Cardiovascular risk: moderate (reference: low) | 1.25 | 0.64 | 2.45 | 0.516 |
| Cardiovascular risk: high (reference: low) | 3.77 | 0.52 | 27.51 | 0.191 |
| Over-the-counter medication, current: physician recommended (reference: not) | 0.64 | 0.36 | 1.13 | 0.127 |
| Functional assessment score, physician-rated | 1.12 | 0.99 | 1.26 | 0.082 |
| Current OA severity, physician-rated: severe (reference: mild) | 2.08 | 0.78 | 5.57 | 0.144 |
| OA severity 6 months ago, patient-reported: moderate (reference: mild) | 1.64 | 0.87 | 3.08 | 0.125 |
| OA severity 6 months ago, patient-reported: severe (reference: mild) | 1.62 | 0.56 | 4.70 | 0.373 |

Multivariate logistic regression, self-managed vs. physician-treated. Physician treatment defined as current prescription of medication/s for OA and/or physician recommendation/s for specified nonpharmacologic treatment for OA (physical or occupational therapy, acupuncture, transcutaneous electrical nerve stimulation, or cognitive behavior therapy/psychotherapy). Self-management defined as no prescription medication or specified nonpharmacologic treatment. The table includes all factors identified as important by LASSO. *p-*value indicates level of significance in the final model. Constant: odds ratio 0.51 (95% CI: 0.11, 2.30). Log pseudolikelihood = –226.31. Number of observations = 751. Wald chi^2^(26) = 156.00, Prob > chi^2^ = 0.00, Pseudo R^2^ = 0.25
^a^Nonpharmacologic therapy requiring no/minimal medical supervision (which were ineligible for the determination of treatment status)

*CI* confidence interval, *LASSO* least absolute shrinkage and selection operator, *OA* osteoarthritis

Fig. S1. Disposition of patients

All patients were categorized according to both physician treatment status and prescription medication status. *Non-Rx* not prescription-medicated, *OA* osteoarthritis, *Rx* prescription-medicated
